# Prevalence of fibromyalgia syndrome in Saudi Arabia: a systematic review and meta-analysis

**DOI:** 10.1186/s12891-023-06821-z

**Published:** 2023-08-30

**Authors:** Yasser Bawazir

**Affiliations:** Department of Medicine, King Abdulaziz University, Jeddah, Saudi Arabia

**Keywords:** Fibromyalgia Syndrome, Meta-analysis, Prevalence, Saudi Arabia, Systematic review

## Abstract

**Background:**

The current systematic review and meta-analysis was conducted to estimate the prevalence of fibromyalgia syndrome (FMS) in Saudi Arabia.

**Methods:**

A PRISMA systematic search appraisal and meta-analysis were conducted. A systematic literature search of English publications in PubMed, Embase, PsycINFO, Web of Science, MEDLINE, and Google Scholar, was conducted up to December 2022. Generic, methodological and statistical data was extracted from the eligible studies. Meta-analysis was done using Comprehensive Meta-Analysis Software. The effect size estimates were calculated using the Fail-Safe N test. The funnel plot, Begg’s and Mazumdar’s rank correlation tests were employed to find any potential bias. The strength of the association between two variables is assessed using Kendall’s tau. A fixed-effects model for the meta-analysis.

**Results:**

The literature search yielded a total of 2479 studies. Eight papers were included in the final analysis with sample size ranging 40 to 1686. All studies were cross-sectional except two, which were retrospective. The total number of the participants recruited in the included articles was 4967 (1794 males and 3173 females); with age ranged between 20 and 79 years. There was high heterogeneity among studies articles (Q = 270.187; p-value 0.001); the tau value was 0.179. The pooled event rates and the 95% confidence intervals (CIs) for the prevalence of FMS in Saudi Arabia in a fixed-effects model was 13.4% (95% CI: 0.124–0.144).

**Conclusion:**

Our results clearly demonstrate that FMS is highly prevalent (13.4%) in Saudi Arabia. It also more common among women. The high prevalence of FMS in Saudi Arabia should be seriously considered and planners should take steps to reduce it.

**Supplementary Information:**

The online version contains supplementary material available at 10.1186/s12891-023-06821-z.

## Introduction

One of the most prevalent musculoskeletal illnesses in adults, particularly women between the ages of 20 and 55, is fibromyalgia syndrome (FMS) [1]. A systemic illness called FMS is defined by the occurrence of lower abdominal pain or cramps, depression, exhaustion, waking up feeling unrefreshed, and chronic widespread musculoskeletal pain [2]. Insomnia and general hypersensitivity are other signs [3]. FMS frequently coexists with other painful illnesses, including temporomandibular joint problems, migraine, interstitial cystitis, and irritable bowel syndrome [4].

FMS is thought to affect 2–4% of people worldwide [5]. Additionally, FMS affects 7.4% of women between the ages of 70 and 79, which is an increase in this condition’s prevalence among women by an 8 to 9-fold rate compared to that of males [1].

Heidari et al. in a meta-analysis showed that the prevalence of FMS among 3,500,756 people of general population was 1.78% [6]. In European Union region the prevalence was varied from 0.29% in the study carried out by Sauer in Germany [7] to 11.10% in the study conducted by Okumus in Turkey [8]. In addition, the total prevalence in the general population of Eastern Mediterranean region, and the Western Pacific general population was 2.41% and 1.60% [6].

FMS is influenced by genetics, environmental factors, and an unknown underlying etiology [2, 9, 10].

According to the evidence that is now available, various studies on the prevalence of FMS have been conducted in Saudi Arabia. Combining the results of these primary studies provides reliable evidences for policymaking to reduce the possible consequences of the syndrome. However, there is limited epidemiological data about the total prevalence of FMS in the general population in Saudi Arabia. Therefore, this study aims to estimate the total prevalence of FMS in Saudi Arabia using systematic review and meta-analysis method.

## Materials and methods

### Protocol registration

The recommended reporting items for systematic reviews and meta-analyses (PRISMA) criteria were followed for conducting the current systematic review and meta-analysis (Appendix table A) [11]. The study protocol was itemized in the International Prospective Register of Systematic Reviews (PROSPERO CRD42023388417).

### Search strategy

To find published articles documenting the prevalence of FMS in Saudi Arabia, we searched the databases of PubMed, Embase, PsycINFO, Web of Science, MEDLINE, and Google Scholar without time limit.

The following is a sample of keywords used during the search process: fibromyalgia, fibromyalgia-fibromyositis, fibromyositis fibromyalgia syndrome, fibromyositis-fibromyalgia syndromes, fibromyositis-fibromyalgia, fibromyalgia, secondary, fibromyalgia, primary fibromyalgia, prevalence, incidence, epidemiology, frequency, Saudi Arabia, Saudi Community, and Saudi Society.

Appendix table B presents a combination of search methods used to find relevant articles in the PubMed database as an example, including (1) search MESH (medical subject heading) and (2) free-text search.

### Eligibility criteria

The prerequisites for inclusion were as follows: published articles mentioned the prevalence of FMS in Saudi Arabia. However, the following exclusion standards were taken into account: reviews, articles written in languages other than English, case reports, studies whose primary goal was not to determine the prevalence of FMS in Saudi Arabia, studies that lacked pertinent information, studies that reported the prevalence of the condition outside of Saudi Arabia, and studies without full texts.

### Study screening and data extraction

The screening operations were controlled using the EndNote V.X8 program, and duplicates were removed. After eliminating duplicates, the author independently evaluated the titles, abstracts, and complete texts to establish the studies’ eligibility. Using a standardized data collection form that was created in accordance with the sequence of variables required from the included articles, the first author’s name and the year of publication, the study setting, the study design, the gender (male or female), the mean age (year), the study participants, the sample size, and the prevalence of FMS in Saudi Arabia were extracted and independently recorded.

### Quality assessment

The 22 methodological items on the STROBE checklist were used to evaluate the included article’ quality [12]. Each article ‘s lowest and highest ratings were 0 and 44. Low quality articles (less than 15.5), moderate quality article (15.5–29.5), and high-quality article s were all assigned to these investigations (30–44).

### Statistical analysis

Software for Comprehensive Meta-Analysis was used to analyze the data (CMA, version 3, BioStat, Tampa, FL, USA). The Fail-Safe N technique was used to estimate the number of studies that should be included in the meta-analysis to recalculate the effect size value obtained. The effect sizes of the studies included in the meta-mean analysis were determined. The information is displayed on forest plots. Using a random-effects model, the prevalence of FMS in Saudi Arabia was compiled and assessed. The event rate, associated 95% confidence intervals, and p-value were all computed using the retrieved data.

In addition to using a funnel plot to analyze publication bias, the included publications were also subjected to Begg’s and Mazumdar’s rank correlation tests to check for any signs of it.

## Results

### Search results

**Fig. 1:**
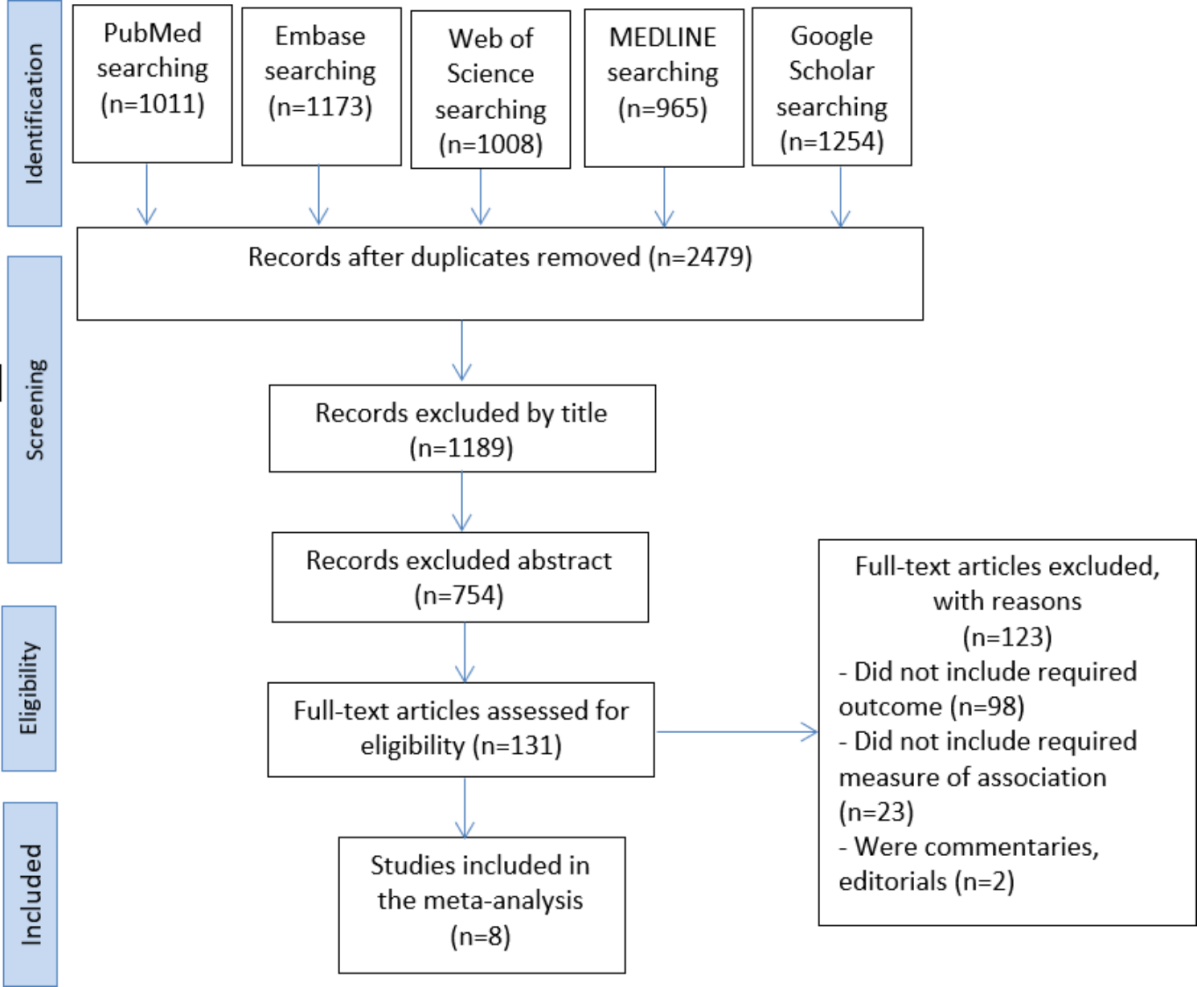
Literature search and review flowchart for selection of articles

After duplicate articles were removed, 2479 articles were left out of the 5411 that were found during the primary search. Reviewing the titles and abstracts revealed 1189 and 754 articles. One hundred twenty-three unrelated articles were found after a full-text review. Ultimately, eight articles [13–20] were found to be eligible for meta-analysis after 123 publications were excluded based on inclusion/exclusion criteria and quality evaluation (Fig. 1).

### Characteristics of the included articles

The overall sample size within the included articles was 4,868 participants (minimum = 40, maximum = 1,686, average = 608.5). Two articles were published in 2022 [15, 16], three articles were published in 2021 [13, 14, 20], one article was published in 2019 [17], one article was published in 2018 [19], and one article was published in 2006 [18]. The studies were conducted in different places in Saudi Arabia, as follows: Taif city [16], outpatient clinics and the daycare unit in a tertiary care hospital in south-western Saudi Arabia [15], different hospitals in Saudi Arabia [14], a non-profit professional Saudi pharmaceutical society [13], King Abdulaziz University [20], the rheumatology department of Al-Ameen Hospital in Taif [17], a single academic institution in Riyadh [19], and a teaching hospital in Jeddah [18]. All studies were cross-sectional in design except two, which were retrospective [15, 18]. The total number of males recruited in the included articles was 1794 (minimum = 8, maximum = 728, average = 224.25), while the total number of females recruited in the included articles was 3173 (minimum = 32, maximum = 958, average = 396.66). The included studies used different tools to collect data on the prevalence of FMS in Saudi Arabia, namely Fibromyalgia Survey Diagnostic Criteria Scale (FSDC), the FiRST test (Fibromyalgia Rapid Screening Tool), London Fibromyalgia Epidemiological Study Screening Questionnaire (LFESSQ), and Fibromyalgia Survey Questionnaire (FSQ). The average prevalence of FMS in Saudi Arabia reported in the included articles was 19.53 (minimum = 7.6, maximum = 48.8) (Table 1).

**Table 1:** Characteristics of the studies included in the meta-analysis

First author name and year of publication	Study setting	Study design	Gender Male/Female	Mean age (year)	Study participants	Sample size	Data collection tool	Prevalence of fibromyalgia (%)
Althobaiti et al. 2022 [16]	Taif city	Cross-sectional	286/729	Age group (20–79)	Taif city residents	1015	FSDC	7.6%
AlOmair et al. 2022 [15]	Outpatient clinics and the daycare unit in a tertiary care hospital in South-Western Saudi Arabia	Retrospective	37/273	Age group (21–70)	seropositive rheumatoid arthritis patients	310	NM	15%
AlEnzi et al. 2021 [14]	Different hospitals in Saudi Arabia	Cross-sectional	408/584	37.3	Health workers	992	FiRST and LFESSQ	16.2%
AlAujan et al. 2021 [13]	Non-profit professional Saudi pharmaceutical society	Cross-sectional	64 /228	29	Pharmaceutical Society	193	FiRST and LFESSQ	48.8%
Samman et al. 2021 [20]	King Abdulaziz University	Cross-sectional	159/291	21.5	Medical students	450	FSDC	9.6%
Amin et al. 2019 [17]	Rheumatology Department of Al-Ameen Hospital in Taif	Cross-sectional	8/32	37.8	Patients diagnosed with FMS	40	NM	42.5%
Omair et al. 2018 [19]	Single academic institution in Riyadh	Cross-sectional	104/78	28	Physicians in training	182	FiRST, LFESSQ and FSQ	8.6%
Kaki 2006 [18]	Teaching hospital in Jeddah	Retrospective	728/958	Age group (50–59)	Chronic pain patients	1686	NM	7.9%

NM denotes Not Mentioned, FSDC denotes Fibromyalgia Survey Diagnostic Criteria Scale, FiRST denotes Fibromyalgia Rapid Screening Tool, LFESSQ denotes London Fibromyalgia Epidemiological Study Screening Questionnaire, and FSQ denotes Fibromyalgia Survey Questionnaire.

### Integrated results

The two statistical models that can be used for a meta-analysis are fixed effect and random effect models [21]. As a result, the fixed effect model only analyzes intra-study sampling errors, whereas the random effect model evaluates both intra-study sampling errors and inter-study variance [22]. The choice of meta-analysis model thus depends on the presence or absence of heterogeneity. If there is no heterogeneity, a fixed effect model is used. However, when there is heterogeneity in the trials, a random effect model should be used [23]. When the study groups are homogenous, both models generate results that are equivalent; however, when the study groups are heterogeneous, the random effect model usually provides wider confidence intervals (CIs) than the fixed effect model [24].

In the eight included papers, the point estimates of the impact size and the 95% confidence interval were 0.134 (95% CI: 0.123–0.144) and 0.158 (95% CI: 0.094–0.252), according to analyses of the fixed and random effects. However, the fixed random model’s homogeneity test (Q-value) suggests that the prevalence of FMS in Saudi Arabia has a heterogeneous structure (Q = 270.187; p-value 0.001). A heterogeneity p-value of 0.10 (rather than 0.05) suggests the presence of heterogeneity since Cochran’s Q test has weak statistical strength and is insensitive [25]. Due to the heterogeneity of the sample, we decided to use a fixed-effects model for the meta-analysis. However, the random-effects model was used to examine the prevalence of FMS. The tau value, which measures actual heterogeneity amongst the included studies, was 0.179 (Table 2).In the eight included papers, the point estimates of the impact size and the 95% confidence interval were 0.134 (95% CI: 0.123–0.144) and 0.158 (95% CI: 0.094–0.252), according to analyses of the fixed and random effects. However, the fixed random model’s homogeneity test (Q-value) suggests that the prevalence of FMS in Saudi Arabia has a heterogeneous structure (Q = 270.187; p-value 0.001). A heterogeneity p-value of 0.10 (rather than 0.05) suggests the presence of heterogeneity since Cochran’s Q test has weak statistical strength and is insensitive [25]. Due to the heterogeneity of the sample, we decided to use a fixed-effects model for the meta-analysis. However, the random-effects model was used to examine the prevalence of FMS. The tau value, which measures actual heterogeneity amongst the included studies, was 0.179 (Table 2).

**Table 2:** Duval and Tweedie’s trim and fill

Model	Effect size and 95% confidence interval			Test of null (2-Tail		
Model	Point of estimate	Lower limit	Upper limit	Z-value	P-value	Q-value
Fixed	0.134	0.123	0.144	-8.370	0.001	270.187
Random	0.158	0.094	0.252	-1.841	0.066	

### Distribution of true effects

**Fig. 2:**
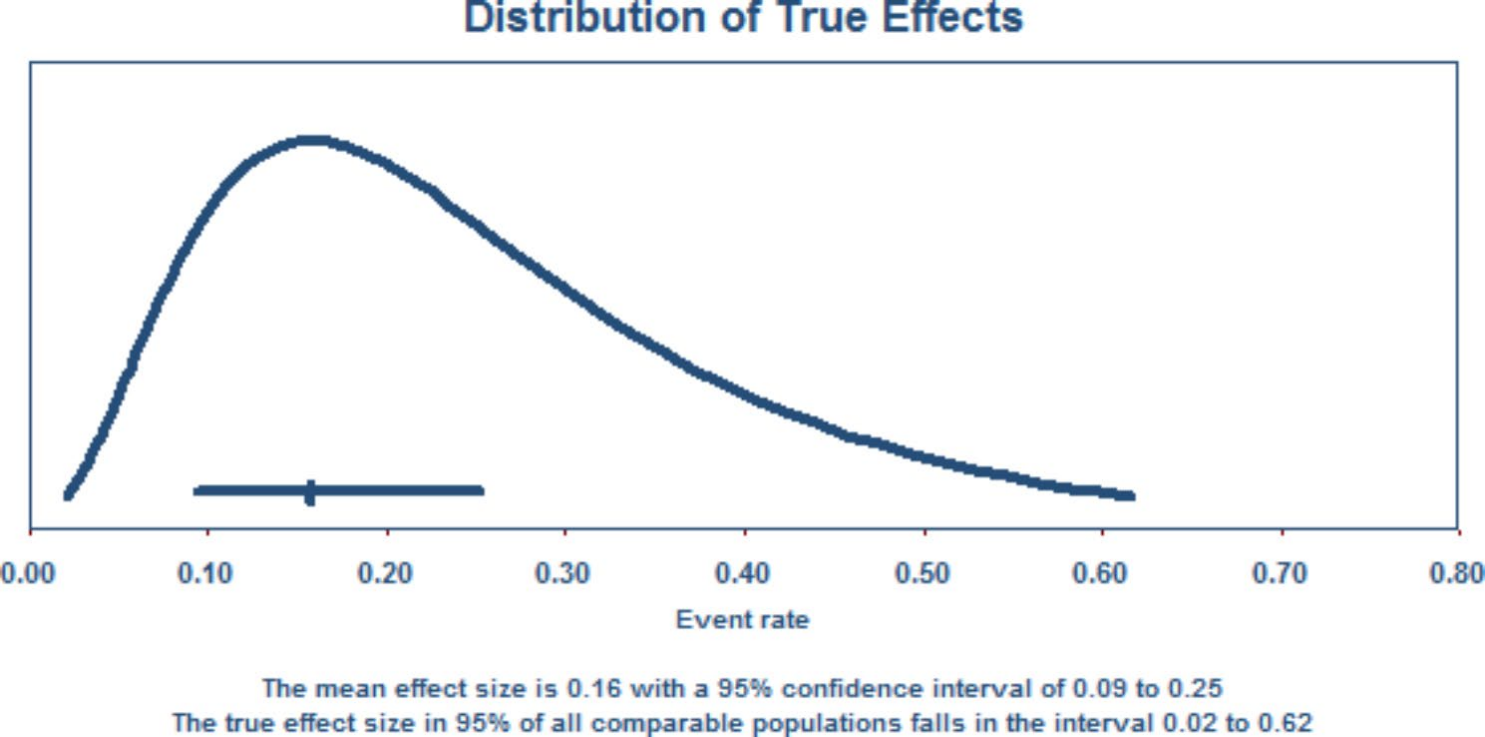
The distribution of true effects

Figure 2 shows that the mean effect size is 0.16, with a 95% confidence interval of 0.09 to 0.35. The true effect size in 95% of all comparable populations falls in the interval of 0.02 to 0.62.

### Orwin’s and classic fail-safe N findings

The Fail-Safe N technique was used to estimate the number of studies that should be included in the meta-analysis to recalculate the effect size value obtained from the meta-analysis [26]. The 2727 (N value), which was obtained using the usual Fail-Safe N approach at a very high level, demonstrates that the effect value generated by our meta-analysis is relatively robust to publication bias (Table 3).

**Table 3:** Orwin’s and classic fail-safe N outcomes

Classic Fail-Safe N Method		Orwin’s Fail-Safe N Method	
-36.234	Z-value for observed studies	0.134	The event rate is observed in studies
0.001	The P-value for observed studies	0.500	The criterion for a “trivial” event rate
0.050	Alpha	0.500	Mean event rate in missing studies
2.000	Tails		
1.959	Z for alphas		
8.000	Number of observed subgroups in the studies		
2727.000	Number of missing studies that would bring the P-value to > alpha (N value)		

### Forest plot for the prevalence of FMS in Saudi Arabia

**Fig. 3:**
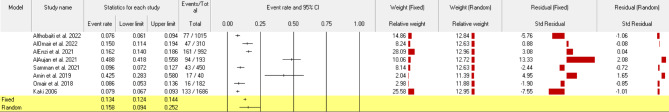
Forest plot for the prevalence of FMS in Saudi Arabia from the fixed and random-effects analysis

The pooled event rates and the 95% confidence intervals (CIs) for the prevalence of FMS in Saudi Arabia in fixed and random models were 0.134 (95% CI: 0.124–0.144) and 0.158 (95% CI: 0.094–0.252) (Fig. 3).

### Rank correlation

The results of the Egger’s regression intercept and the rank correlation by Begg and Mazumdar test did not reveal any conclusive indication of a significant publishing bias. Kendall’s tau with and without continuity as well as Egger’s regression intercept’s two-tailed p-values were 0.179, 0.214, and 0.491 (Table 4).

**Table 4:** Egger’s regression intercept and the Begg and Mazumdar rank correction

Kendall’s S statistic (P-Q)	6.000
Kendall’s tau with continuity correction	
Tau	0.179
z-value for tau	0.619
P-value (1-tailed)	0.269
P-value (2-tailed)	0.536
Kendall’s tau without continuity correction	
Tau	0.214
z-value for tau	0.742
P-value (1-tailed)	0.229
P-value (2-tailed)	0.458
Egger’s regression intercept	
Intercept	4.495
Standard error	6.132
95% low limit (2-tailed)	-10.511
95% upper limit (2-tailed)	19.499
t-value	0.448
Df	6.000
*P*-value (1-tailed)	0.246
*P*-value (2-tailed)	0.491

### Publication bias

**Fig. 4:**
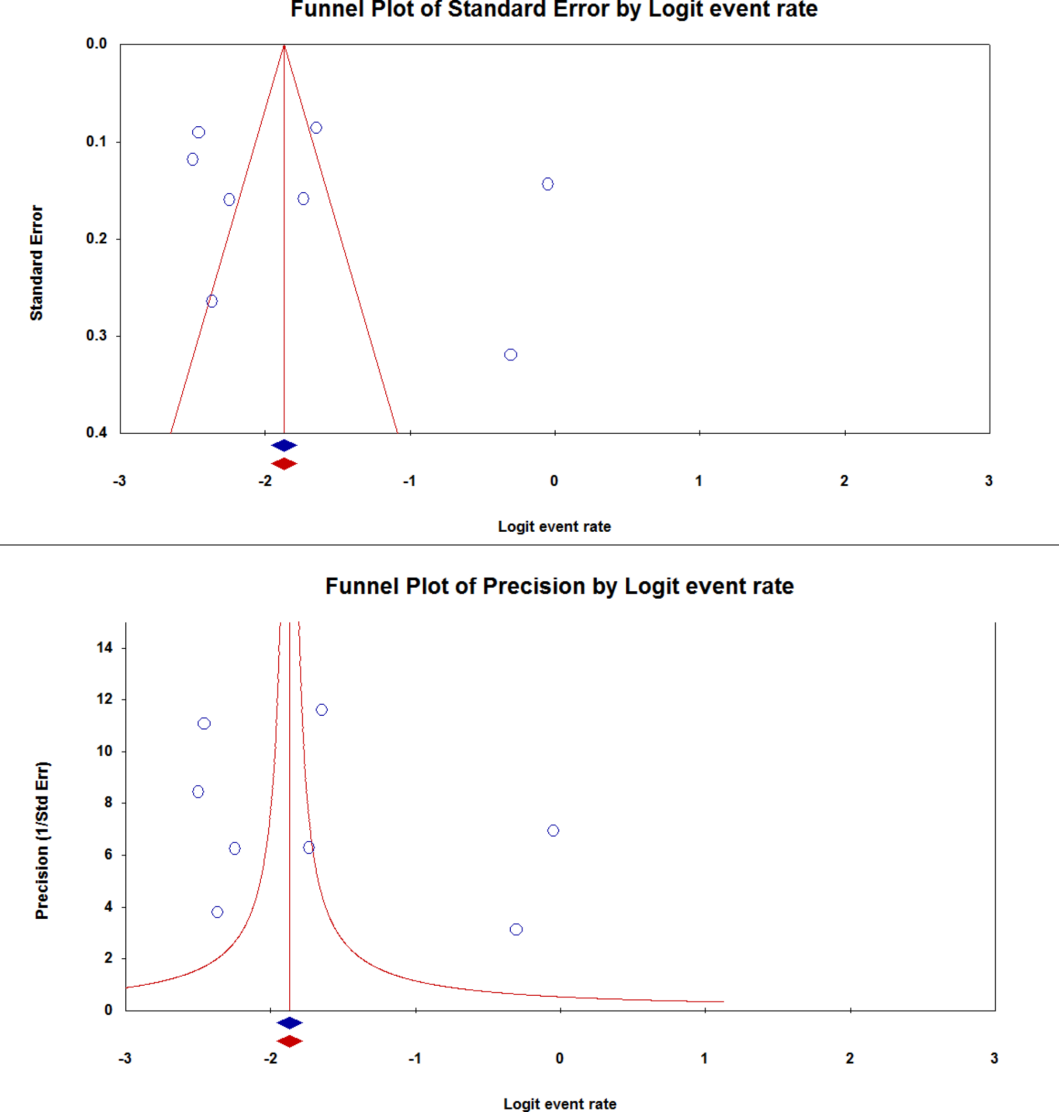
Publication bias for the prevalence of FMS in Saudi Arabia

The funnel plot of publication bias for the prevalence of FMS in Saudi Arabia is shown in Fig. 4.

### Subgroup analysis

**Fig. 5:**
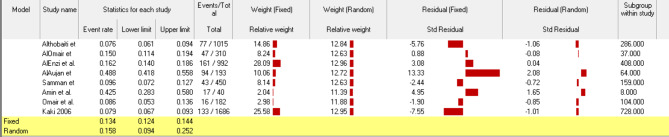
Pooled prevalence of FMS in Saudi Arabia according to male participants

Figure 5 shows the forest plot for the prevalence of FMS in Saudi Arabia from the fixed and random-effects analysis according to male participants. The results of the current systematic review and meta-analysis demonstrated that females were affected with FMS more than males (3173 females vs. 1794 males).

## Discussion

The current systematic review and meta-analysis was conducted to estimate the prevalence of FMS in Saudi Arabia. To the author’s knowledge, the present systematic review and meta-analysis is the first to demonstrate the prevalence of FMS in Saudi Arabia. In this systematic review and meta-analysis, after fixed-effects model meta-analysis, results showed that the prevalence of FMS in Saudi Arabia is 13.4%. There was a considerable heterogeneity among the included studies. In the current systematic review and meta-analysis, a systematic literature search of six databases yielded a total of 2479 studies. Eight papers were included in the final analysis with sample size ranging 40 to 1686. All studies were cross-sectional in design except two, which were retrospective. The total number of the participants recruited in the included articles was 4967 (1794 males and 3173 females); with age ranged between 20 and 79 years.

The prevalence of FMS was reported to be 1.60% among 3081 French people over the age of 18 in the study conducted by Perrot et al. [27]. According to a research by Lindell et al., which involved 147 Swedish citizens, the prevalence of FMS and persistent generalized pain was 1.30 and 4.20% [28]. Furthermore, among 522 patients hospitalized in the internal department, a cross-sectional study by Buskila et al. found that 15% of patients had FMS, and 91% of these patients were women [29].

In addition, the prevalence of FMS reported in the included studies was ranged from 7.6 to 48.8% this can be explained by different diagnostic techniques, disparities in the classifications utilized, and variances in the populations studied may all contribute to the high frequency, differences of the prevalence of FMS in Saudi Arabia [30]. Furthermore, individuals from various ethnic groups have been found to have this illness in the majority of nations with diverse climates [31].

However, the current systematic review and meta-findings analysis’s showed that more women than men were affected by the FMS (3173 females vs. 1794 males). According to Croft et al., the prevalence of FMS ranged from 0.2 to 3.9% and from 0.7 to 13% for men and women [32]. Another study came to the conclusion that women are 8–9 times more likely than men to have FMS. Our study’s findings confirm these conclusions [1].

In contrast, the included research used several diagnostic criteria and instruments, including FSDC, FiRST, LFESSQ, and FSQ, to gather information on the prevalence of FMS in Saudi Arabia. A patient-administered questionnaire called the FSDC evaluates diagnosis and symptom severity. The FSDC is a reliable and constructively valid tool for FMS patients. It has the potential to establish itself as the gold standard for measuring polysymptomatic distress in FMS since it is simple to use, quick to complete, and easy to score [33]. Additionally, the FiRST instrument is a quick, easy, and uncomplicated self-administered questionnaire with excellent discriminative value that may be useful for FMS detection in both clinical research and everyday practice [33]. Besides, general population surveys of non-institutionalized individuals seem to benefit from using the LFESSQ method to screen for FMS [31]. The FSQ questionnaire was also a reliable tool for use in survey research among people with fibromyalgia and chronic pain problems [34].

Arnold et al. (2019) have suggested a different method for making the FMS diagnosis while taking into account the limitations of the prior the American College of Rheumatology diagnostic criteria [35, 36]. The Analgesic, Anaesthetic, and Addiction Clinical Trial Translations Innovations Opportunities and Networks (ACTTION), a public-private partnership with the American Pain Society (APS) and the Food and Drug Administration (FDA), developed the ACTTION-APS Pain Taxonomy (AAPT) to create a clinically useful and uniform diagnostic system for chronic pain disorders, including FMS. To create new diagnostic criteria for FMS, the AAPT created an international working group of doctors and academics with expertise in the condition [37].

In fact, the high prevalence of FMS in the current systematic review and meta-analysis also may be related to differences in the definitions and tools used to identify the prevalence of the syndrome. Therefore, the use of a single and valid criterion would identify the prevalence of FMS with greater sensitivity.

The current systematic review and meta-analysis is prone to some limitations including the high heterogeneity among studies articles. In addition, using different diagnostic methods is other limitations for our study. The strength of this meta-analysis is that it is the first study, which shows the overall prevalence of FMS in Saudi Arabia. The present systematic review and meta-analysis study may provide a baseline data on the prevalence of FMS in Saudi Arabia, which can be a suitable opportunity for researchers and policymakers to reduce the possible consequences of the syndrome.

## Conclusions

Our results clearly demonstrate that FMS is highly prevalent (13.4%) in Saudi Arabia; It also more common among women. The high prevalence of FMS in Saudi Arabia should be seriously considered and planners should take steps to reduce it. Further future studies using single and more accurate diagnostic criteria and represent the exact sampling tools and characteristics of the study population are recommended.

### Electronic supplementary material

Below is the link to the electronic supplementary material.

Supplementary Material 1

## Data Availability

The data presented in this study are available on request from the corresponding author.
